# Brugada Phenocopy due to Hyponatremia: A Case Report and Review of the Literature

**DOI:** 10.19102/icrm.2025.16026

**Published:** 2025-02-15

**Authors:** Christos S. Konstantinou, Dimitrios Sfairopoulos, Konstantinos Zekios, Konstantinos P. Letsas, Panagiotis Korantzopoulos

**Affiliations:** 1 First Department of Cardiology, University of Ioannina Medical School, Ioannina, Greece; 2 Arrhythmia Unit, Onassis Cardiac Surgery Center, Athens, Greece

**Keywords:** Brugada phenocopy, Brugada syndrome, electrolyte abnormalities, hyponatremia

## Abstract

Brugada phenocopy (BrP) is the electrocardiographic appearance of a Brugada pattern due to various reversible causes that is completely resolved after the correction of the underlying abnormalities. In this short communication, we describe a 56-year-old man who had a transient BrP induced by hyponatremia due to thiazide diuretic therapy. A detailed review of the literature revealed that hyponatremia represents an uncommon cause of BrP while, in many of the published cases, concomitant electrolyte disturbances such as hyperkalemia were present. However, even isolated hyponatremia may provoke a BrP. Clinicians should be aware of this rare cause of BrP, which is reversible and has a favorable outcome.

## Introduction

Brugada syndrome (BrS) represents an inherited channelopathy, associated with a risk for sudden cardiac death, diagnosed when a coved-type ST-segment elevation is present in leads V1 and/or V2 placed in the standard or higher positions.^1,2^ Given that BrS is usually asymptomatic, the diagnosis is often incidental in patients investigated for other medical conditions. Moreover, it is not always evident in the electrocardiogram (ECG) and can be unmasked by various conditions, such as fever or administration of class I anti-arrhythmic drugs. However, several reversible clinical conditions, such as myocardial ischemia, electrolyte abnormalities, pulmonary embolism, right ventricular mechanical compression, and others, may provoke transient electrocardiographic abnormalities mimicking BrS. This phenomenon is termed Brugada phenocopy (BrP) and should be differentiated from BrS.^3,4^

## Case presentation

**Figure 1:**
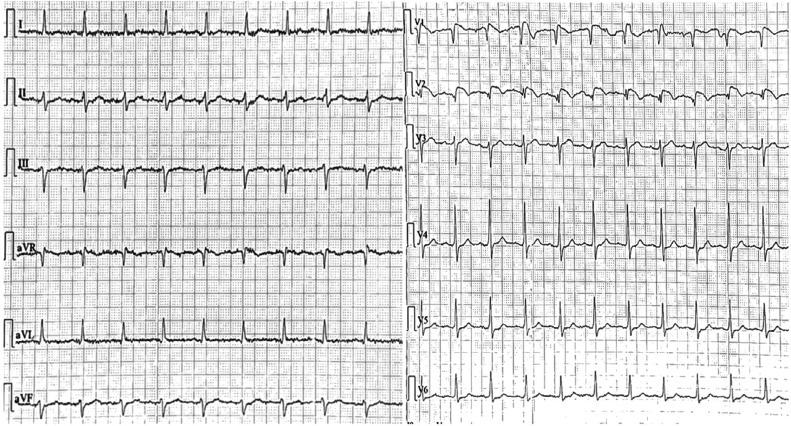
The patient’s 12-lead electrocardiogram on admission.

A 56-year-old man presented to the emergency department of our hospital complaining of general weakness and numbness in the lower extremities. His past medical history was significant only for hypertension treated with a fixed combination of irbesartan and hydrochlorothiazide (150 + 12.5 mg/day). This medication was initiated 15 days before, while the patient was taking only irbesartan until this time point. No other acute medical illness was noted at his presentation. The clinical examination was unremarkable, while the core body temperature was 36.5°C. He denied any history of palpitations or syncopal episodes. Also, his family history was unremarkable. Of note, the blood biochemical tests showed hyponatremia (Na^+^: 125 mEq/L) and marginal potassium levels (K^+^: 3.5 mEq/L), while magnesium levels were normal (Mg^2+^: 2 mg/dL). The rest of the blood laboratory tests, including troponin and thyroid function tests, were all within normal limits. Interestingly, a 12-lead ECG on admission showed concave ST-segment elevation and negative T-waves in leads V1 and V2, consistent with the Brugada type I pattern **(Figure 1)**. Despite the presence of some artifacts in lead V1, lead V2 was more consistent with the type I Brugada pattern **(Figure 1)**. No specific cause of hyponatremia was evident apart from the diuretic use.

**Figure 2:**
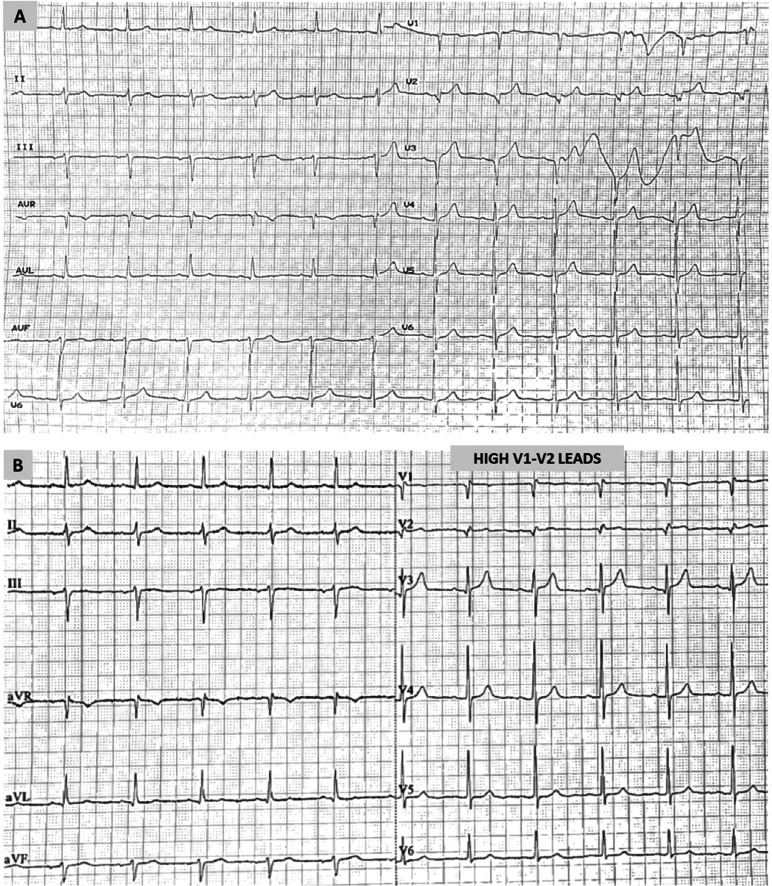
**A:** The 12-lead electrocardiogram on the fifth day of hospitalization with V1–V2 leads on standard positions. **B:** The 12-lead electrocardiogram on the fifth day of hospitalization with V1–V2 leads to higher positions over the second intercostal space.

**Figure 3:**
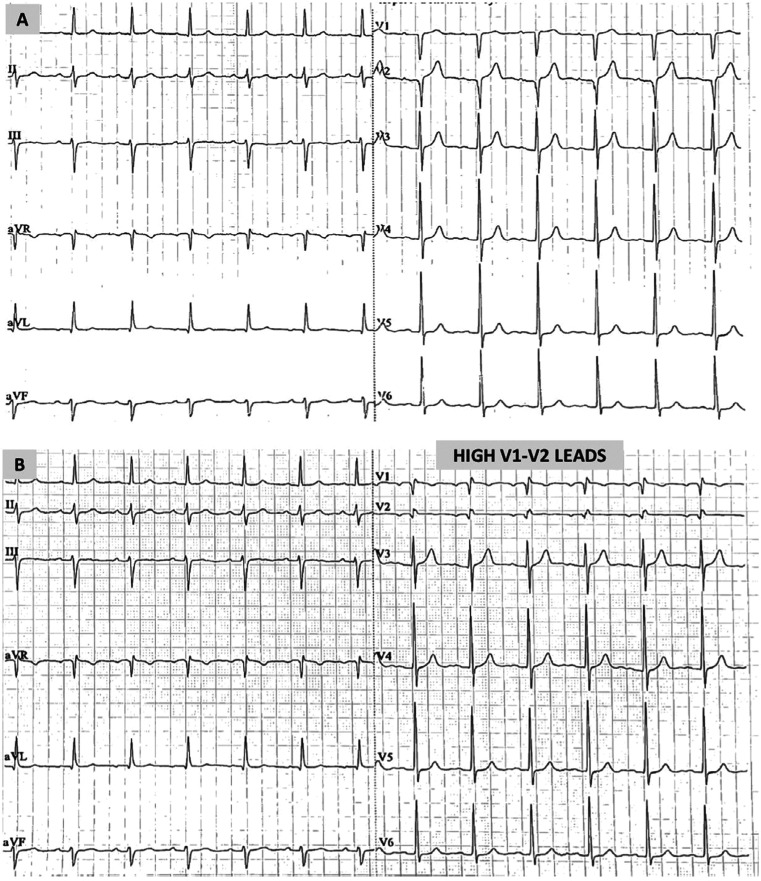
**A:** The 12-lead electrocardiogram 6 months after hospitalization with V1–V2 leads on standard positions. **B:** The 12-lead electrocardiogram 6 months after hospitalization with V1–V2 leads moved to higher positions over the second intercostal space.

The patient was admitted to our department, and his anti-hypertensive medication was replaced by valsartan 160 mg plus amlodipine 5 mg. An echocardiogram and a myocardial perfusion scan (single photon emission computed tomography) showed no significant abnormalities. On the fifth day of hospitalization, the ECG completely normalized, with no ST-segment elevation in leads V1 and V2, even in high precordial positions (second intercostal space) **(Figures 2A and 2B)**. This normalization became evident, along with the resolution of hyponatremia. Specifically, on the fifth day of hospitalization, serum Na^+^ was 137 mEq/L and serum K^+^ was 3.9 mEq/L. Moreover, a drug provocative test using procainamide (10 mg/kg, infusion for 10 min) did not reveal any abnormality. The patient was discharged the following day in excellent clinical condition. Six months after the index hospitalization, he remains asymptomatic, having a normal ECG with no ST-segment elevation in V1–V2, even in high precordial positions **(Figures 3A and 3B)**, and normal serum electrolyte levels receiving valsartan and amlodipine.

The patient gave signed consent for publication of his medical case anonymously and without showing his face. The report has been approved by the local institutional ethics committee.

## Discussion and literature review

BrP is considered a relatively benign electrocardiographic phenomenon that should be carefully recognized and characterized since the diagnosis of BrS has a different therapeutic approach. Remarkably, patients exhibiting BrP have reversible underlying conditions. Therefore, BrS and BrP cannot be differentiated by their ECG patterns. In BrP, there is a clear reversible underlying condition, and thus the ECG abnormality is transient. However, the exact mechanisms and the prognosis of BrP remain elusive. Transient abnormalities in ion currents induced by various factors seem to underlie the observed electrocardiographic alterations that resemble the type I Brugada pattern.^3,4^ Correspondingly to the depolarization and repolarization theories of BrS, transient conditions that cause transmural gradients in the right ventricular outflow tract (RVOT) due to the different distribution of transient outward K_+_ current (I_to_) current or delayed depolarization of the RVOT causing conduction delay and creating an electrical gradient may account for the electrocardiographic abnormalities associated with BrP. It is believed that genetic abnormalities of cardiac ion channels are not present in cases of BrP. However, subtle genetic variants predisposing to BrP^3^ or some shared with BrS ionic alterations^4^ cannot be ruled out. Besides, electrolyte abnormalities that may facilitate such electrical disturbances, inflammatory conditions such as pericarditis/myocarditis, ischemia or coronary artery spasm (especially in the right coronary artery), mechanical compression (eg, tumors), pressure overload, and stretch causing mechanoelectrical disturbances (eg, pulmonary embolism) in the RV or the RVOT area have been associated with BrP. Bearing in mind that all the aforementioned transient abnormalities may facilitate the unmasking of BrS, after resolution of the Brugada pattern, a negative sodium channel blocker provocative test is obligatory to establish the BrP diagnosis.^3,4^

Regarding metabolic and electrolyte disturbances, hyperkalemia, hypokalemia, and hypercalcemia are the most frequently reported causes of BrP.^3^ Hyponatremia is a not-so-common cause of BrP that is increasingly reported in the literature.^5–17^ A systematic review of the literature revealed seven published cases of BrP induced by hyponatremia without other extra causes **(Table 1)**.^5–10^ The most common causes included psychogenic polydipsia and diuretic therapy **(Table 1)**. In another eight published cases of hyponatremia-induced BrP, there were concomitant abnormalities that contributed to the manifestation of BrP, most commonly hyperkalemia.^11–17^ Almost all patients exhibited the Brugada I pattern at presentation **(Tables 1 and 2)**. Of note, no previous publication reported an ECG with high precordial leads after resolution of the electrocardiographic abnormalities. Moreover, in only two cases, a drug provocation test was performed after the normalization of the ECG.^11,12^ It should also be noted that, in the second case by Agrawal and colleagues,^7^ the ECG does not show a clear Brugada pattern. Additionally, in the study by Ayad and colleagues,^16^ the initial ECG mentioned to exhibit the Brugada pattern is not included in the article.

**Table 1:** Cases of Brugada Phenocopy Induced by Hyponatremia Without Additional Causes

Study	Patient Age (Years), Sex	Main Symptoms	Past Medical History	Brugada Pattern	Sodium Level at Presentation	Cause of Hyponatremia	Drug Challenge Test—Result	Resolution After Correction of Hyponatremia
Tamene et al., 2010^5^	63, male	Generalized weakness, drowsy mental status	Hypertension, diabetes mellitus, dyslipidemia, bipolar disorder	Type I	101 mEq/L	Psychogenic polydipsia, thiazide diuretic, poor dietary salt intake	No (the patient refused)	Yes
Alvarez et al., 2011^6^	30, female	Edema anasarca, oliguria	None	Type I	121 mEq/L	Nephrotic syndrome	No (the patient refused)	Yes
Agrawal et al., 2016,^7^ Case 1	63, female	Confusion, altered mental status	Diabetes mellitus, hypertension, schizoaffective disorder	Type I	112 mEq/L	Psychogenic polydipsia	No	Yes
Agrawal et al., 2016,^7^ Case 2^a^	54, male	Lethargy, vomiting, anorexia, decreased fluid intake	Hypertension	Type II (?)	106 mEq/L	Dehydration?	No	Yes
Ramsaroop, 2019^8^	49, male	Acute delirium	Hypertension	Type I	108 mEq/L	Thiazide diuretic	No (the patient refused)	Yes
Rattanawong and Senthong, 2021^9^	73, male	Epigastric pain, nausea, vomiting, breathlessness, palpitations	Diabetes, hypertension, epilepsy	Type I	126 mEq/L	Possibly due to gabapentin intake	No	Yes
Yilmaz and Özdemir, 2023^10^	84, male	Weakness, nausea, sleepiness, loss of appetite, diarrhea, confusion	Hypertension, dementia, benign prostatic hyperplasia, COPD	Type I	109 mEq/L	Hypovolemic hyponatremia due to gastrointestinal loss and oral intake disorder	No (the patient refused)	Yes
Present case	56, male	General weakness and numbness in the lower extremities	Hypertension	Type I	125 mEq/L	Thiazide diuretic	Yes—negative	Yes

*Abbreviation:* COPD, chronic obstructive pulmonary disease. ^a^Case 2 in this report did not show a clear Brugada pattern in the electrocardiogram.

**Table 2:** Cases of Brugada Phenocopy Induced by Hyponatremia and Other Concomitant Abnormalities

Study	Patient Age (Years), Sex	Main Symptoms	Past Medical History	Brugada Pattern	Sodium Level at Presentation	Cause of Hyponatremia	Concomitant Abnormalities	Drug Challenge Test—Result	Resolution After Correction of Hyponatremia and Concomitant Abnormalities
Kovacic and Kuchar, 2004^11^	38, male	Vomiting, diarrhea, lethargy	None	Type I	105 mEq/L	Diabetic ketoacidosis	Hyperkalemia (7 mEq/L), acidosis	Yes—negative	Yes
Mok et al., 2008^12^	64, male	Generalized weakness and dizziness	Hypertension	Type I	111 mEq/L	Diuretic agent (indapamide)	Hypokalemia (1.7 mEq/L)	Yes—negative	Yes
Hunuk et al., 2016^13^	61, male	Lethargy, impaired level of consciousness	Recent prostate surgery for benign hyperplasia	Type I, and type II	124 mEq/L	Acute postrenal failure	Hyperkalemia (7.1 mEq/L)	No (the patient refused)	Yes
Alanzalon et al., 2018,^14^ Case 1	18, male	Nausea, vomiting, diarrhea	Diabetes mellitus type I	Type I	120 mEq/L	Diabetic ketoacidosis	Hyperkalemia (7.7 mEq/L), acidosis	No	Yes
Alanzalon et al., 2018,^14^ Case 2	7, male	Nausea, vomiting, polyuria	None	Type I	124 mEq/L	Diabetic ketoacidosis	Acidosis	No	Yes
Landa et al., 2021^15^	48, male	Diffuse pain, fatigue, nausea, vomiting	Diabetes mellitus type I	Type I	122 mEq/L	Diabetic ketoacidosis	Hyperkalemia (7.6 mEq/L), acidosis	No	Yes
Ayad et al., 2021^16,a^	58, male	Pleuritic chest pain, cough, fever, chills, thirst, increased urination	Old pulmonary tuberculosis	Type I (?)	116 mEq/L	Serious febrile infection—pneumonia	Severe SARS-CoV-2 infection—the patient died	No	Yes
Amusina et al., 2022^17^	26, male	Weakness, palpitations, syncope, nausea, vomiting, diarrhea	Attention-deficit hyperactivity disorder	Type I, and type II	94 mEq/L	Primary adrenal insufficiency (Addison’s disease)	Hyperkalemia (6.1 mEq/L), hypochloremia	No	Yes

*Abbreviations:* ECG, electrocardiogram; SARS-CoV-2, severe acute respiratory syndrome coronavirus 2. ^a^This publication does not report the initial electrocardiogram mentioned to show a type I Brugada pattern.

The available literature suggests that, in most cases, the BrP is benign, but such may depend on the underlying condition, while relative prospective data are very few.^3,4,18^ In the setting of hyperkalemia, the study by Xu et al. showed that hyperkalemia-induced BrP is not associated with sudden cardiac death or ventricular arrhythmias.^19^ However, in another study, malignant arrhythmias in the setting of hyperkalemia were more frequent in those who had a concomitant Brugada pattern, indicating a high-risk group.^20^ Also, malignant arrhythmias may occur in cases of BrP in the setting of drug intoxication.^21^ It could be speculated that the presence of BrP may be related to the severity of the underlying condition that obviously should be promptly and effectively treated. In other words, BrP patients do not develop life-threatening ventricular arrhythmias in the long term, and therefore implantable cardioverter-defibrillators should not be implanted.

In this case report, our patient had no other concomitant abnormality, and no structural heart disease was evident. In our patient, complete resolution of the Brugada type I pattern coincided with the correction of the sodium levels. The cessation of the offending agent (hydrochlorothiazide) was sufficient for the effective management of hyponatremia and led to the resolution of the electrocardiographic abnormalities. Indeed, drug-induced hyponatremia is a common clinical problem, while diuretics are the most common culprit agents.^22^ Moreover, the subsequent negative provocative test using a sodium channel blocker and the absence of Brugada pattern recurrence after correction of hyponatremia point against the diagnosis of BrS and favor a BrP phenomenon. ECG recordings, even in high V1–V2 leads, 6 months after the index event did not demonstrate a Brugada pattern. It should be pointed out that drug challenge provocative tests have limited sensitivity for the diagnosis of BrS.^1,2^ Genetic testing is also non-conclusive in several cases, and it is not ideal even for familial screening.^1,2^ Of note, no genetic testing was performed in any of the published cases of hyponatremia-associated BrP.

## Conclusion

Hyponatremia is a rare cause of BrP and should be included in its potential causes. Clinicians should be aware of the BrP, a reversible and benign condition with a favorable outcome in most instances. A misleading diagnosis of BrS may lead to unnecessary diagnostic procedures and false management plans that may have an adverse impact on patients’ outcomes.
